# Targeted therapies for gastric cancer: failures and hopes from clinical trials

**DOI:** 10.18632/oncotarget.14825

**Published:** 2017-01-26

**Authors:** Maria Apicella, Simona Corso, Silvia Giordano

**Affiliations:** ^1^ Department of Oncology, University of Torino, Candiolo Cancer Institute-FPO, IRCCS, Candiolo, Torino, Italy

**Keywords:** gastric cancer clinical trials, targeted therapies, HER2, tyrosine kinase receptors, immunotherapy

## Abstract

Gastric cancer is the third leading cause of cancer mortality worldwide. As surgery is the only curative treatment strategy and conventional chemotherapy has shown limited efficacy -with a median overall survival of 10 months- new treatments are urgently needed. Trastuzumab and Ramucirumab (targeting HER2 and VEGFR2, respectively) are the only targeted therapies approved so far. Indeed, most Phase III clinical trials evaluating molecular drugs in gastric cancer failed. This review will retrace the relevant clinical trials with molecular therapies performed in gastric cancer patients, discussing the possible reasons for their failure and indicating new perspective for a real improvement of the treatment of this disease.

## INTRODUCTION

Gastric cancer is the third leading cause of global cancer-related deaths worldwide [1], with approximately one million new cases diagnosed each year. In spite of the significant advances in surgical techniques, improvements in diagnosis and development of new chemotherapy protocols, the overall clinical outcome for patients with advanced gastric cancer is poor, with 5-20% 5-year survival and 10 months median overall survival (OS) [2]. Incidence is strongly influenced by ethnical and geographical factors, and it is higher in Eastern Asia, Eastern Europe, and South America, while North America and Africa show the lowest recorded rates [3]. About 80-90% of gastric carcinomas develop in a sporadic setting, the remaining 10% to 20% show familial cluster, and approximately 1-3% have a clear inherited genetic susceptibility [4].

Two main classifications are in use to define gastric adenocarcinomas. The WHO (World Health Organization) classification describes four histological subtypes (papillary, tubular, mucinous and poorly cohesive) while Lauren’s classification identifies intestinal, diffuse, or mixed subtypes [5]. However, these two classifications do not have a prognostic value and cannot help guide the therapeutic approach. Very recently, two wide molecular classifications have been published (Table 1). The Cancer Genome Atlas Group identified four major molecular subtypes [6]: i) the most frequent (around 50% of tumors) is characterized by chromosomal instability (CIN) and amplification of genes, mainly encoding tyrosine kinase receptors; ii) tumors with Microsatellite Instability (MSI, 22%), presenting a very high mutation rate and DNA methylation; iii) genomically stable (20%) tumors and iv) Epstein Barr Virus positive tumors (9%), characterized by DNA hypermethylation, high frequency of PIK3CA mutations and PDL1/PDL2 overexpression. Even if this classification has no prognostic value, as no survival difference was found among the four subgroups, it represents a milestone for the identification of new molecular targets and the design of new therapeutic approaches.

**Table 1:** Molecular classifications of gastric cancer proposed by The Cancer Genome Atlas and the Asian Cancer Research Group

TCGA (The Cancer Genome Atlas)		ACRG (Asian Cancer Research Group)	
SUBTYPES	MOLECULAR FEATURES	SUBTYPES	MOLECULAR FEATURES
EBV (9%)	-DNA hypermethylation -high frequency of PIK3CA mutations -PDL1/PDL2 overexpression	MSS/TP53+ (26%)	-frequent EBV positivity -intermediate mutation rate
MSI (22%)	-high mutation rate -DNA methylation	MSI (23%)	-high mutation rate
GS (20%)	-molecular alterations in cell adhesion/ cell migration pathways -ARID1 and BCOR mutations	EMT (15%)	-low mutation rate -loss of epithelial markers
CIN (50%)	- chromosomal instability (CIN) -amplification of genes (most encoding tyrosine kinase receptors)	MSS/TP53- (36%)	-TP53 mutations -genomic instability

EBV = Epstein Barr Virus; MSI = Micro Satellite Instable; MSS = Micro Satellite Stable; EMT = Epithelial Mesenchymal Transition; GS = Genomically Stable; CIN = Chromosomal Instability.

A new gastric cancer classification based on molecular characteristics was provided also by the Asian Cancer Research Group [7]. They also identified a MSI group, while the MSS (Microsatellite Stable) tumors were subdivided on the basis of evidence of epithelial-mesenchymal transition (EMT), presence of wild type TP53 or TP53 inactivation. Interestingly, these molecular subtypes were associated with clinical parameters. The MSS/EMT was characterized by the worst prognosis and a higher chance of recurrence, while the MSI subtype showed the best prognosis.

Altogether, these two classifications represent a critical forward step in our understanding of the molecular basis of gastric cancer and can be crucial to develop rational molecular therapies to improve the outcome of these patients. For the moment, in fact, the main therapeutic options rely on surgery and the use of cytotoxic drugs such as platinum-based agents, irinotecan and taxanes. Differently from other tumors where many therapeutic options are available on the basis of the molecular characteristics of the tumor, only two target therapies have been approved by FDA (*Food and Drug Administration, USA)* and European Union for advanced gastric cancer (Trastuzumab -October 2010- and Ramucirumab -December 2014) but most of the patients, at the moment, do not get a benefit from them.

## HER2 IN GASTRIC CANCER

**Figure 1:**
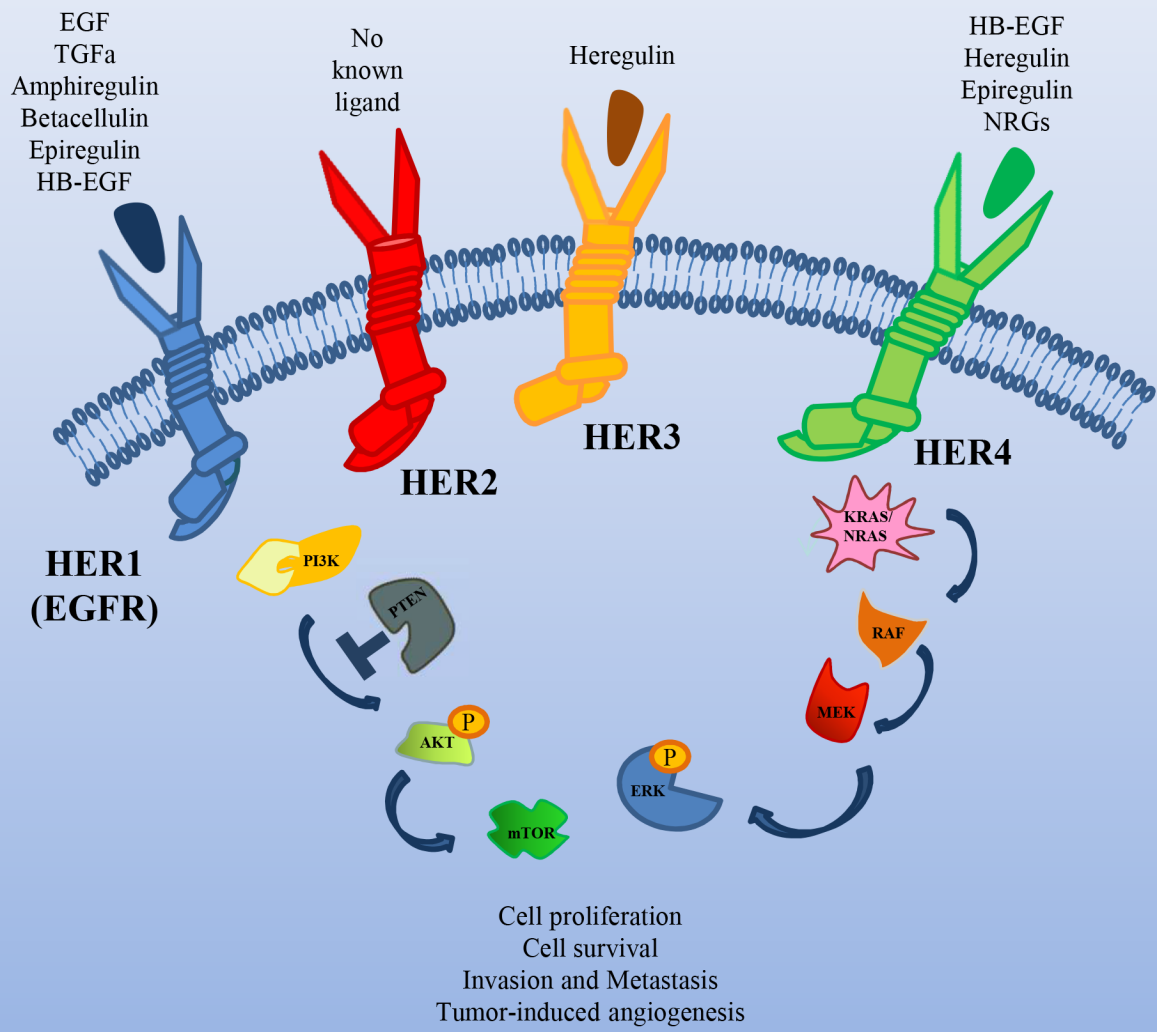
The HER family of Receptor Tyrosine Kinases Schematic illustration of the receptors of this family, their ligands and their major downstream signal transducers.

The HER2 receptor is a member of the Epidermal Growth Factor Receptor (EGFR) family, with the unique property of lacking a high affinity specific ligand (Figure 1). Its activation is due to its spontaneous homo/heterodimerization with the other EGFR family receptors, of which it is the preferred dimerization partner [8]. HER2 is amplified in different tumors, such as breast, pancreatic, colorectal and gastric cancer [9]. In particular, in breast and colon cancer, HER2 amplification correlates with response to anti-HER2 drugs [10].

**Figure 2:**
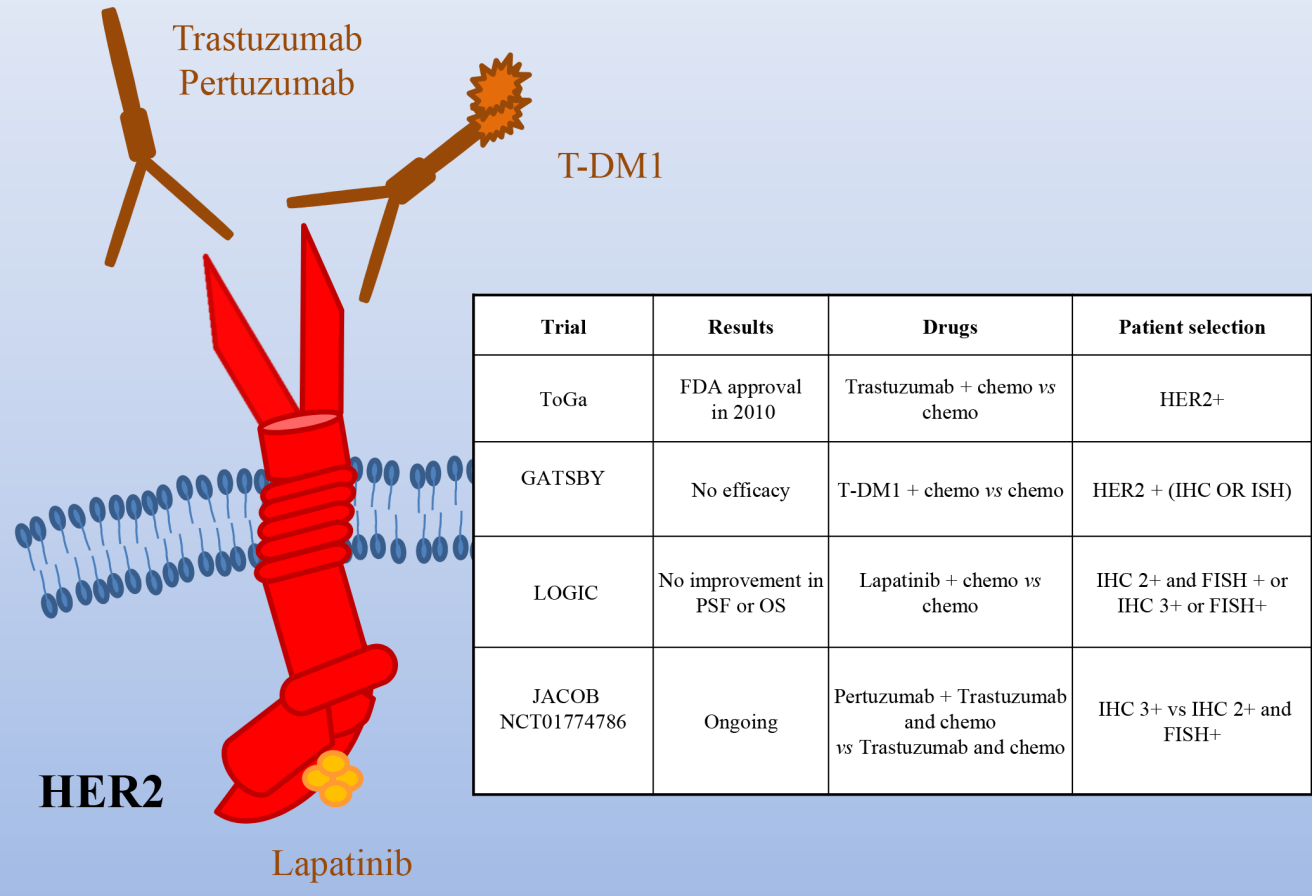
HER2 as a target in gastric cancer Schematic illustration of the HER2 receptor and of the targeted drugs tested in clinical trials (the mAbs Pertuzumab and Trastuzumab -and its emtansine conjugated, T-DM1- and the dual HER1/HER2 small kinase inhibitor Lapatinib). The insert table shows the major trials targeting HER2. PFS = Progression Free Survival; OS = Overall Survival.

Several studies showed HER2 overexpression and amplification in gastric cancer, but due to difference in the examination methods, the frequency of positivity is considerably different in the diverse studies, ranging from 6% to 30% [11–19]. Notably, gastric cancer frequently shows heterogeneity of the HER2 genotype and phenotype that can be partially responsible for testing inaccuracy [20]. *HER2* testing in gastric cancer reveals important differences compared to breast cancer: i) in breast, full membrane staining is mandatory for 2+/3+ evaluation, while in gastric cancer lateral or basal staining is sufficient; ii) differently from breast cancer, gastric cancer often stains heterogeneously; thereof, a threshold of 10% positive cells was considered appropriate to assess HER2 status (30% for breast tumors). Despite this consensus, discordances are still present concerning the best criteria to determine HER2 positivity. In fact, while EMEA (European Medicines Agency) has recommended IHC (immunohistochemistry) as initial screening, with 3+ samples considered positive and 2+ positive if confirmed by FISH (Fluorescence in situ hybridization) analysis (Annex I. Summary of product characteristics. European Medicines Agency; 2010), FDA recommended selection of either 3+ or FISH+ patients. In light of the relatively high frequency of overexpression/amplification of HER2 in gastric cancer, preclinical and early phase clinical studies have been performed to evaluate the therapeutic potential of its targeting in this context [10, 21, 22]. Phase II trials [23, 24] provided the rationale for the ToGA study, evaluating Trastuzumab plus chemotherapy (capecitabine, cisplatin) versus chemotherapy alone in HER2+ advanced gastric/gastroesophageal patients [25] (Figure 2). The investigators analyzed patients through both IHC and FISH and randomized 594 HER2+ cases; less than half of them were classified as FISH positive, IHC 3+. Overall survival, the primary endpoint of the study, was significantly longer in patients receiving Trastuzumab plus chemotherapy (13·8 months *vs* 11·1; HR: 0·74; 95% CI 0·60-0·91). Progression free survival (6·7 *vs* 5·5 months), overall response rate (47% *vs* 35%) and the duration of response (6·9 *vs* 4·8 months) were increased as well in patients receiving Trastuzumab. Subgroup analysis showed that OS was significantly extended in patients with higher HER2 expression (IHC 3+ or IHC 2+/FISH positive) treated with Trastuzumab (16 *vs* 11.8 months). Conversely, in 131 patients with low HER2 expression, the addition of Trastuzumab did not improve survival. Altogether these results suggest that Trastuzumab provides the best therapeutic benefit to strongly HER2+ patients. Similar results have been recently obtained in the non-interventional study HERMES [26].

Although the results of the ToGA trial led to the approval of Trastuzumab in HER2+ metastatic gastric patients, there are still open questions. It is not clear if the addition of Trastuzumab to standard chemotherapy is effective in the adjuvant setting after previous gastrectomy as the 133 patients falling in this category did not show statistical improvement when treated with Trastuzumab. It is not clear as well if patients with locally advanced disease could benefit from this therapy, as only 20 of them were analyzed and no improvement was observed upon Trastuzumab. Finally, no stratification was done according to the site of gastric origin or the pathology of the disease, so it is unclear if these factors have predictive value. In light of the new knowledge, it will be interesting to evaluate if patients with HER2 amplification of the EBV subtype behave as those of the CIN subtype and if patients with HER2 mutations, found mainly in the MSI subtype, are also responsive to the treatment.

Another open question is the opportunity to continue Trastuzumab in second line therapy beyond progression. To evaluate this point in gastric cancer patients a study was retrospectively performed in patients progressed after first-line Trastuzumab-based chemotherapy [27]. Thirty two HER2+ gastric cancer patients (IHC 3+ or IHC 2+/FISH+) received fluoropyrimidine-, platinum- and Trastuzumab-containing regimens as first-line therapy and irinotecan or taxanes with or without Trastuzumab as second-line therapy. No significant difference in PFS (median, 3·6 *vs*. 2·8 months) and OS (median, 9·5 *vs*. 7·6 months) was observed between the two cohorts, while marginal improvements in PFS (median, 4·4 *vs*. 2·1 months) and OS (median, 7·8 *vs*. 6·7 months) were observed when confined to the IHC 3+ subgroup. Larger prospective trials are required to definitely prove that continuing Trastuzumab monotherapy until disease progression could extend overall survival in these patients.

Recently, T-DM1, which combines Trastuzumab with the anti-microtubule agent emtansine, has been approved for HER2+ breast cancer. Preclinical works have shown that T-DM1 is active in gastric cancer models [28, 29]. Unfortunately, a randomized, open-label, phase II/III study of T-DM1 versus a taxane in patients with previously treated HER2+ locally advanced or metastatic gastroesophageal tumors (GATSBY) did not show efficacy benefit over taxanes [30]. The reasons of this negative result are not clear. As expected, T-DM1 performed better in HER2 IHC3+ patients than in negative ones. However, almost no difference in outcome was observed in patients previously treated with HER2-targeted therapies *vs*. naïve patients. Thus, it is unlikely that a problem of resistance due to previous treatments can justify the negative results. A possible explanation is that systemic chemotherapy plus Trastuzumab performs better than T-DM1 because the former can take care also of low-level/HER2- clones. Alternatively, or in addition, as it up to 35% of gastric tumors lost their HER2+ status after I line therapy, a decreased level of HER2 could impair the activity of T-DM1 but not that of taxanes. Lapatinib is a dual EGFR/HER2 small kinase inhibitor approved for treatment of HER2+, locally advanced or metastatic breast cancer [31]. The Southwest Oncology Group performed a phase II trial of Lapatinib as first line therapy in patients with advanced or metastatic gastric cancer [32]. The study (47 patients not selected for HER2 amplification) showed only a modest single-agent activity. The LOGIC trial, a double-blinded study, investigated Lapatinib in combination with capecitabine plus oxaliplatin in HER2+ advanced or metastatic first line gastroesophageal carcinoma patients [33]. The addition of Lapatinib did not significantly increase OS. A phase III study (TyTAN) was performed in HER2 amplified advanced gastric cancer Asian patients, treated with paclitaxel alone or in association with Lapatinib in second line [34]. Median OS was 11·0 months with Lapatinib plus paclitaxel versus 8·9 months with paclitaxel alone (*P* = 0·1044), with no significant difference in median PFS (5·4 *vs* 4·4 months) or TTP (5·5 *vs* 4·4 months). Better efficacy with Lapatinib plus paclitaxel was demonstrated in IHC 3+ patients. Overall, Lapatinib plus paclitaxel demonstrated activity in second-line treatment but did not significantly improve OS. Very recently, Lorenzen et al. conducted a phase II trial in which 37 pts were randomized to Lapatinib plus capecitabine or capecitabine alone but the study was closed prematurely for futility [35]. Overall, these studies suggest that Lapatinib, as single targeted therapy, is poorly active in gastric cancer. One possible explanation for the different effect of Trastuzumab and Lapatinib in gastric cancer patients might rely on the contribution of antibody-dependent cell-mediated cytotoxicity (ADCC), lacking in the small molecule therapeutic approach. Further studies are needed to evaluate if, as shown in preclinical setting [36], it synergizes with Trastuzumab.

Pertuzumab is a HER2 monoclonal antibody that interferes with HER2 heterodimerization with other EGFR family members. As it is effective in HER2+ breast cancer patients in combination with Trastuzumab [37], a phase III trial (JACOB) is ongoing, in which HER2+ metastatic gastric cancer patients will receive Pertuzumab or placebo, in combination with Trastuzumab and chemotherapy. Randomization will be stratified by region, prior gastrectomy, and HER2-positivity (IHC 3+ *vs* IHC 2+ and ISH+ [in situ hybridization]).

Altogether, these results suggest that HER2 is a good target in gastric cancer even though the criteria to select patients that could benefit from therapy should be better defined. As suggested by Gomez-Martin and colleagues, it is likely that a real “addiction” of cancer cells to HER2 and response to anti-HER2 therapies require a level of amplification of around 10 copies, with a better response in patients showing higher levels of amplification [38]. Moreover, it has yet to be defined which is the best therapeutic strategy as the response to anti-HER2 drugs is somehow “organ specific”; in fact, while Trastuzumab is effective in monotherapy in breast cancer, this is not true in HER2-amplified colon cancer, as demonstrated in Patient-Derived Xenografts (PDXs) preclinical models [39]. However, treatment against HER2 proved to be effective in colorectal cancer patients by using the combination of Trastuzumab and Lapatinib [40]. It will thus be important to determine the best therapeutic approach in gastric patients.

## EPIDERMAL GROWTH FACTOR RECEPTOR

**Figure 3:**
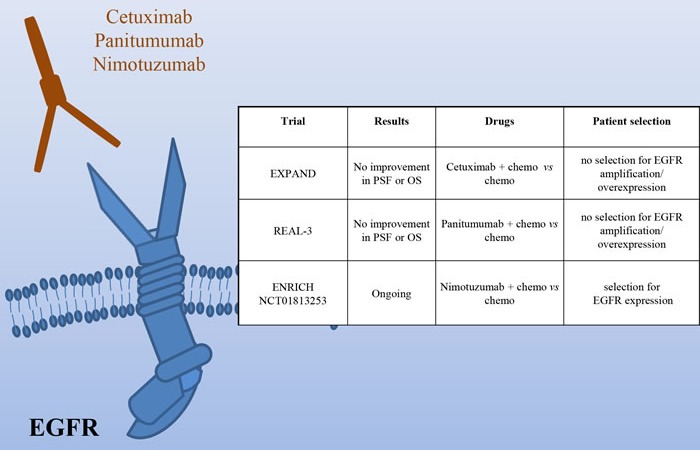
HER1 (EGFR) as a target in gastric cancer Schematic illustration of the EGFR receptor and of the targeted drugs tested in clinical trials (the mAbs Cetuximab, Panitumumab and Nimotuzumab). The insert table shows the major trials targeting EGFR. PFS = Progression Free Survival; OS = Overall Survival.

The Epidermal Growth Factor Receptor is amplified in around 5% of gastric cancers, characterized by poor prognosis [6]. Two phase III studies have investigated the efficacy of Cetuximab in association with chemotherapy (Figure 3). The EXPAND trial, evaluating capecitabine and cisplatin with or without Cetuximab in patients with previously untreated tumors, did not observe any benefit with the addition of Cetuximab [41]. The REAL3 trial, studying epirubicin, oxaliplatin and capecitabine with or without Panitumumab in first line reached similar results [42]. The major problem of these two studies is that no patient selection was done on the basis of EGFR amplification/overexpression. This renders the results quite questionable. In fact, while in colon cancer the EGFR status is not usually analyzed as the wild type receptor activity is considered critical for tumor growth, no such data are available in gastric cancer. Experimental data, indeed, have shown a positive correlation between Cetuximab response and EGFR high expression/amplification [43]. On this line are the results of a phase II trial assessing Cetuximab plus oxaliplatin/leucovorin/5-fluorouracil, showing an association between higher EGFR copy number (≥4) and OS [44]. In addition, trials of novel EGFR agents are ongoing. Of note, the phase III ENRICH trial of irinotecan with or without Nimotuzumab in preselected patients with high EGFR expression.

Overall, before disregarding EGFR as a possible target in gastric cancer, we should carefully identify patients with EGFR pathway activation and evaluate the effect of inhibition of this receptor in this context.

## HEPATOCYTE GROWTH FACTOR/HEPATOCYTE GROWTH FACTOR RECEPTOR

The most frequent genetic alteration of the Hepatocyte Growth Factor Receptor (encoded by the MET gene) is gene amplification, which is associated with shorter survival [45, 46].

**Figure 4:**
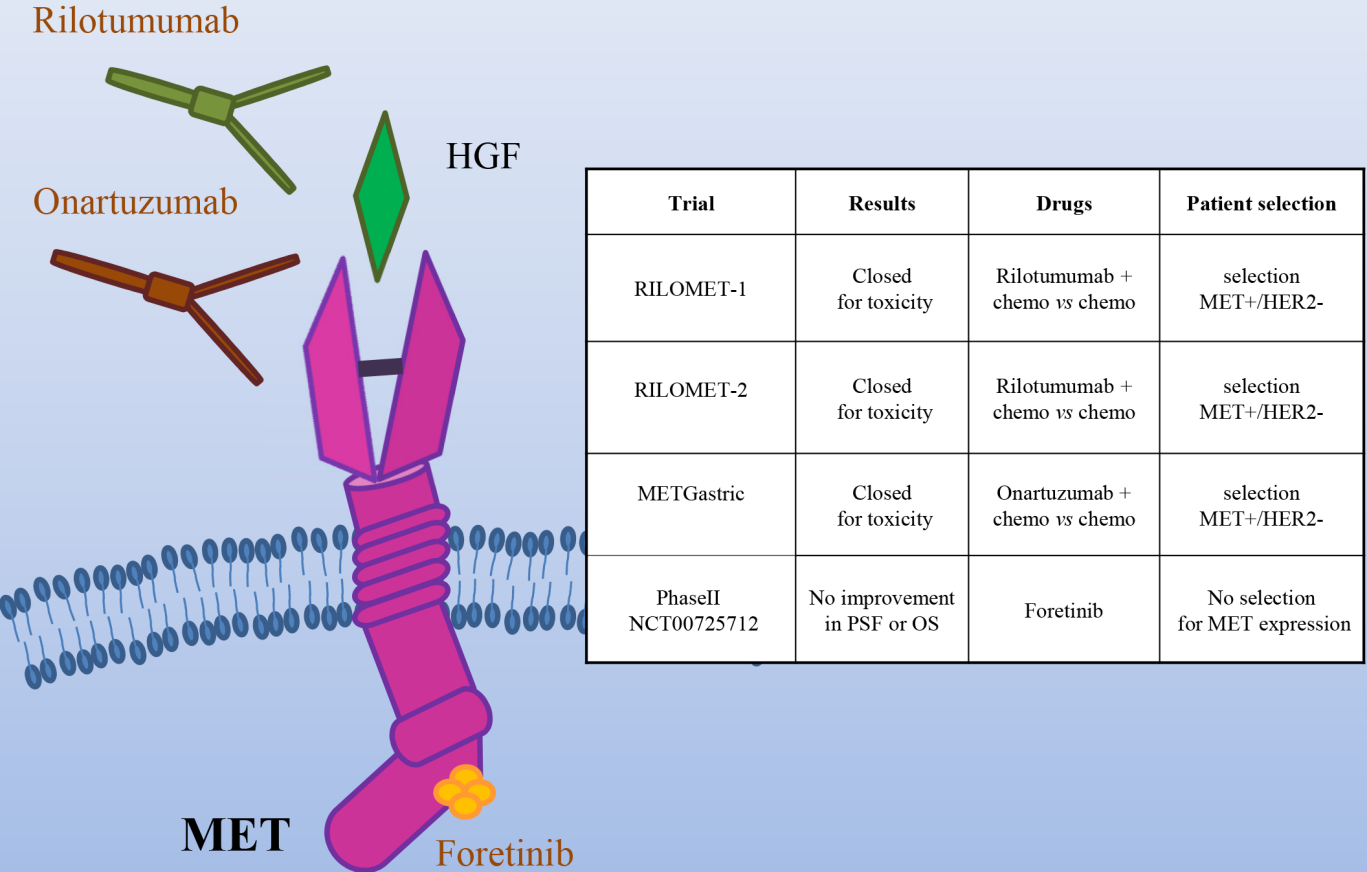
The MET/HGF pathway as a target in gastric cancer Schematic illustration of the MET tyrosine kinase receptor, its ligand HGF and the targeted drugs tested in clinical trials (the HGF mAb Rilotumumab, the MET mAb Onartuzumab and the multikinase small kinase inhibitor Foretinib). The insert table shows the major trials targeting the MET/HGF pathway. PFS = Progression Free Survival; OS = Overall Survival.

In the last years, several drugs targeting the MET/HGF axis have been developed, including monoclonal antibodies (directed against either HGF or MET) and MET small kinase inhibitors (Figure 4). On the basis of a Phase II trial showing a survival advantage from the addition of the anti-HGF antibody Rilotumumab to chemotherapy, two phase III clinical trials were started (RILOMET-1 and RILOMET-2). Unfortunately, both the studies have been prematurely closed, due to an increase in the number of deaths in the Rilotumumab arms [47, 48]. Onartuzumab, a MET antibody, was evaluated in the phase III METGastric trial, in combination with mFOLFOX6 (modified Folinic Acid (Leucovorin)-Fluorouracil-Oxaliplatin 6), in HER2-/MET+ gastric cancer patients. As preliminary results revealed a higher rate of serious toxicities in the experimental arm, in particular in high MET patients, the trial was stopped [49]. Negative results were obtained also in a phase II trial with the multikinase MET inhibitor Foretinib, in molecularly unselected metastatic patients [50]. In spite of the encouraging pre-clinical data, the results of most of the trials targeting MET in gastric cancer are very disappointing, raising doubts about the utility of targeting this oncogene in gastric cancer. It is actually possible that MET is not a good target in this context. However, there were scattered reports of complete and exceptionally durable responses in gastric cancer patients treated with anti-MET antibodies [51] or small molecules [52]. In particular, positive results were reported from a Phase I Trial with the MET specific small molecule AMG 337 (NCT01253707). Among 10 patients bearing MET-amplified gastric and esophageal cancers, one experienced a durable complete response (100 weeks), and four displayed a partial response [52]. One possible explanation for the trial failures so far could be that these trials have not appropriately selected the patients that could benefit from the therapy. The MET gene is amplified in not more than 4% of gastric cancer [6]; moreover, experimental data have shown that tumor cells become “addicted” to MET (and thus responsive to its inhibition) when they have at least 8 gene copies [53]. However, in the studies performed in MET+ patients, the selection has usually been conducted on the basis of immunohistochemistry, that does not discriminate between overexpression and amplification. Notably, MET overexpression in the absence of amplification (e.g. as a consequence of hypoxia [54]) is extremely frequent in cancers. Other possible causes of primary resistance to anti MET drugs in gastric cancer are i) coamplification of different driver oncogenes, a phenomenon that was recently reported to frequently occur in gastro-esophageal cancer and ii) the extensive heterogeneity in MET gene amplification among distinct tumor lesions within the same patient [55]. If this is true, the success of a clinical trial with anti-MET compounds would require a much stringent selection of the patients, based on genomic data on the levels of MET amplification, possibly integrated by comprehensive molecular analysis; moreover, since the molecular profiling of a single-lesion biopsy may be insufficient to guide targeted therapy selection, liquid biopsy strategies might help to better select patients that could really benefit from MET targeted therapies. Finally, very recently, exon 14 skip mutations of MET have been found to confer high sensitivity to MET inhibitors [56, 57]. As such mutations have been identified also in gastric cancer patients [58], it will be very important to evaluate their sensitivity to MET inhibitors. More work is thus required to better identify potentially responsive patients and to evaluate the most effective inhibitors.

## VASCULAR ENDOTHELIAL GROWTH FACTOR/VASCULAR ENDOTHELIAL GROWTH FACTOR RECEPTOR

VEGF/VEGFR2-dependent signaling plays an important role in tumor angiogenesis. Even though at the moment there is no predictive factor for selection of patients that could benefit from this therapy, it has been reported that in gastric cancer VEGF expression and serum levels correlate with more advanced stage disease and poor outcome [59]. Moreover, preclinical data have shown that VEGFR2 inhibition impaired tumor growth and angiogenesis in gastric cancer animal models [60].

**Figure 5:**
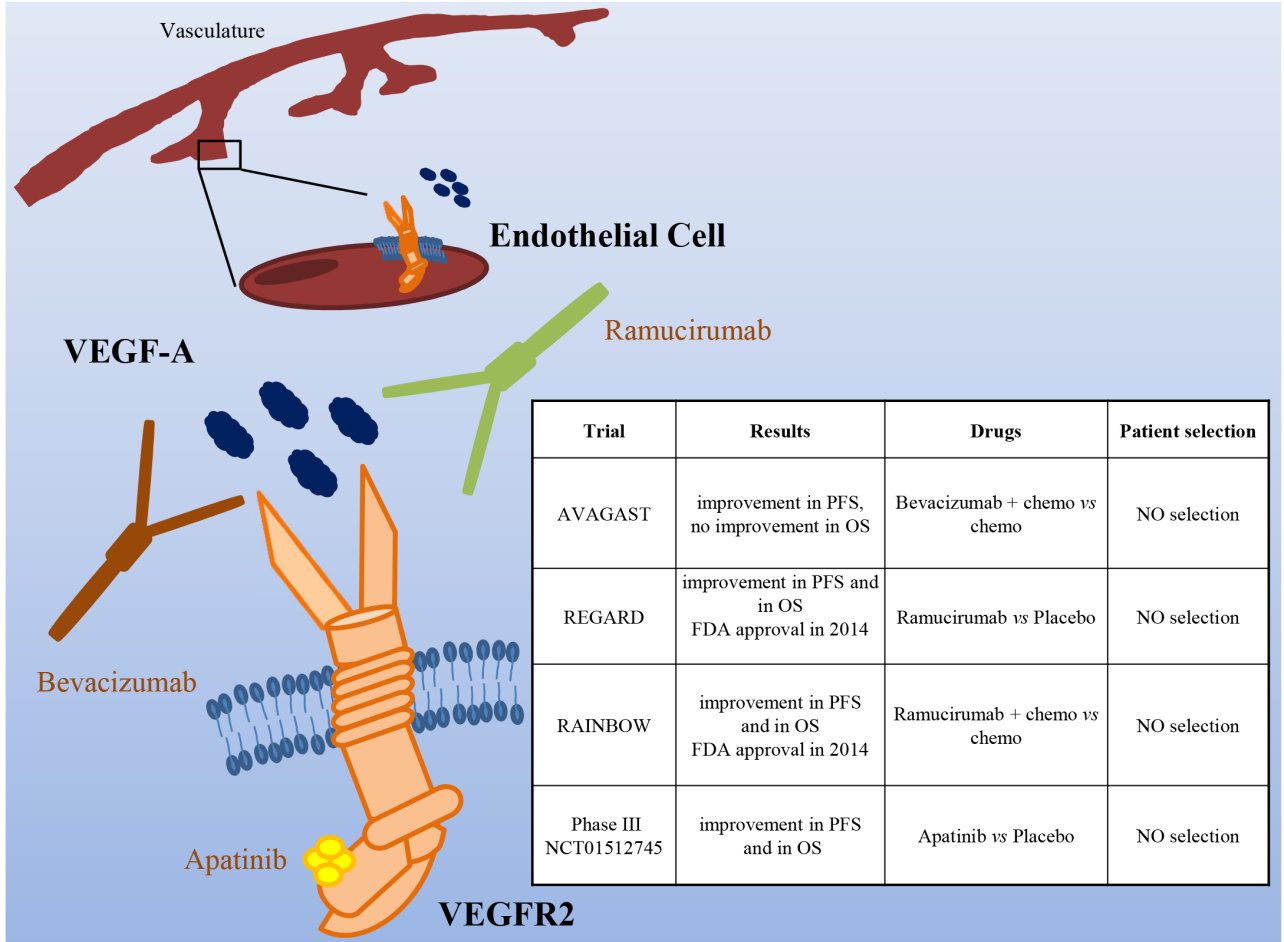
The VEGF/VEGFR pathway as a target in gastric cancer Schematic illustration of VGFR2 (expressed mainly on endothelial cells), its ligand VEGF-A, and targeted drugs tested in clinical trials (the VEGF-A mAb Bevacizumab, the VEGFR2 mAb Ramucirumab and the VEGFR2 small kinase inhibitor Apatinib). The insert table shows the major trials targeting the VEGF/VEGFR pathway. PFS = Progression Free Survival; OS = Overall Survival.

Based on promising results of two phase II studies [61, 62], a phase III trial comparing chemotherapy with or without Bevacizumab (a VEGF-A mAb) in first line gastric cancer patients was conducted [63] (Figure 5). The results showed a significant improvement in PFS (6·7 *vs* 5·3 months) and overall RR (46 *vs* 37·4%), even though the primary endpoint (OS) was not reached. Moreover, the study showed important differences between Western and Asian patients, the formers benefitting most from the anti-angiogenic therapy.

Very recently, two Phase III studies evaluated the role of Ramucirumab, a VEGFR-2 mAb interfering with VEGFs binding to their receptor. The REGARD study evaluated Ramucirumab as second line therapy after disease progression on a first line chemotherapy regimen, in patients with advanced, unresectable gastroesophageal tumors [64]. Median overall survival was 5.2 months in the Ramucirumab group and 3.8 months in the placebo group (*P* = 0·047). A longer PFS (2.1 months for Ramucirumab *vs*. 1.3 for placebo) was also reported. Overall, this study identified Ramucirumab as the first biological treatment given as a single drug showing survival benefits in patients with advanced gastro-esophageal adenocarcinomas progressed after first-line chemotherapy. A second phase III study (RAINBOW) investigated the same antibody in association with paclitaxel, as second line treatment in patients with metastatic gastric cancer who progressed after a first line chemotherapy [65]. Overall survival was significantly longer in the Ramucirumab plus paclitaxel group than in the placebo group (9.6 *vs*. 7.4 months). Furthermore, Ramucirumab plus paclitaxel significantly delayed disease progression (PFS 4.4 *vs*. 2.9 months) and increased response rate (28% *vs*. 16%). Based on these results, Ramucirumab has been approved by FDA and the European Commission either as a single agent or in association with paclitaxel in patients with advanced or metastatic gastric and gastroesophageal junction cancer after progression on fluoropirimidine or platinum containing regimens.

In contrast to the refractory setting, the addition of Ramucirumab was not superior to chemotherapy alone in the first-line setting. Yoon and colleagues, in fact, performed a phase II trial, where 168 patients were randomized to mFOLFOX6 plus Ramucirumab or placebo [66]. Although patients in the investigational arm experienced a higher disease control rate (85% *vs*. 67%), no difference was observed in PFS (6.4 *vs*. 6.7 months) and OS (11.7 *vs*. 11.5 months).

Among the antiangiogenic agents under investigation in gastric cancer is Apatinib, an oral VEGFR-2 inhibitor. In a phase III trial, patients progressed on second line therapy were randomized to Apatinib or placebo. Median OS (140 days with placebo *vs*. 195 days with Apatinib) and PFS (53 *vs*. 78 days) were significantly improved [67].

Although anti-angiogenic therapies have obtained successful results in big trials such as REGARD and RAINBOW, at the moment, for this kind of therapy, there are no biomarker predictors of response. It is thus impossible to select patients with higher probability of response to treatment. The TCGA classification showed that the CIN and Genomically Stable subtypes were associated with VEGF-A gene amplification and elevated expression of angiogenesis-related pathways, respectively. Even though these data are now not sufficient to influence the therapeutic strategies, they can give hints to select patients.

Another consideration stemming from the performed studies is that drugs directed against VEGFR-2 seem to be more effective than those targeting VEGF-A. The reason of this behavior is not clear but it is possible that during tumor evolution neoplastic cells change the expression of the VEGF ligands, thus decreasing the response to VEGF-A targeting drugs.

## OTHER TARGETS

The Fibroblast Growth Factor 2 receptor tyrosine kinase (FGFR-2) is amplified in around 10% of gastric tumors, mainly of the CIN subtype [6] and its amplification is associated with lymphatic invasion and worse prognosis [68]. As preclinical data suggest that FGFR2-amplified gastric cancers respond to targeted inhibitors [69], clinical trials where patients selected for FGFR2 amplification or polisomy are treated with inhibitors such as Dovitinib (NCT01719549) or AZD4547 (NCT01457846) are ongoing.

The PI3K/AKT/mTOR pathway is very frequently altered in human tumors and its activation often sustains resistance to targeted therapies. Mutations of PIK3CA (the PI3K encoding gene) are present in 24% of gastric cancers, being particularly frequent in the EBV and the MSI subtypes (72% and 42%, respectively [6]). Moreover, mutations of PTEN, a negative controller of the PI3K/AKT pathway, are present in 11% of the tumors, more frequently in the MSI subtype. Even though the activation of this pathway is frequent in gastric tumors, a phase III study evaluating the mTOR (a downstream effector of the pathway) inhibitor Everolimus on patients with advanced gastric cancer failed to show improved survival [70]. Similarly, a phase II study of MK-2206, an allosteric inhibitor of AKT, gave negative results [71]. Notably, in neither study enrolled patients were selected for PI3K pathway activation.

Altogether, the results of these trials are not conclusive as treatment was not performed in patients selected to display mutations of target genes and activation of the pathway. More accurate studies are thus required to better understand if PI3K/AKT/mTOR are indeed “drivers” in some gastric cancer and to define the most efficient drugs.

## IMMUNOTHERAPY

**Figure 6:**
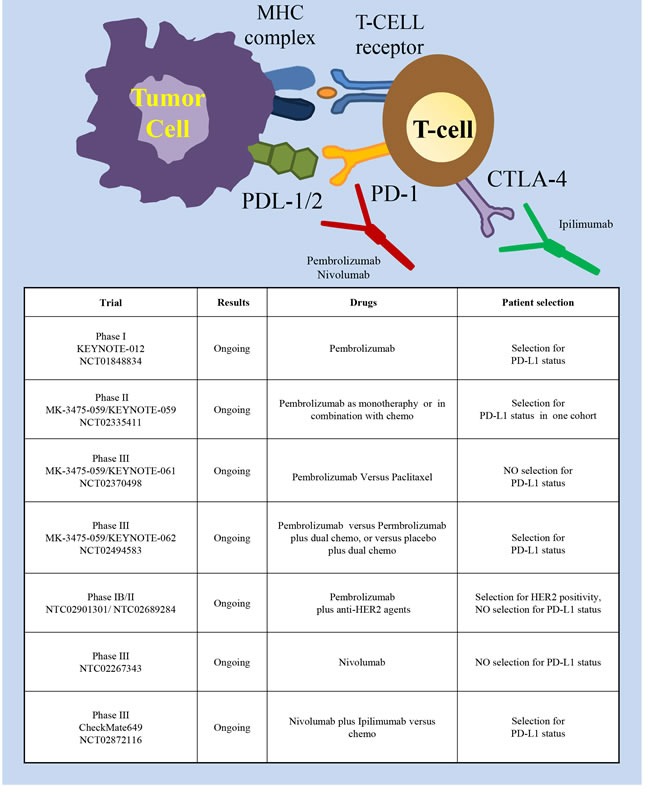
Immune checkpoint inhibitory pathways as targets in gastric cancer Schematic illustration of molecules expressed in tumor cells (left part) and T lymphocytes (right part) involved in the immune checkpoint inhibitory pathway and of the targeted drugs (the PD-1 mAbs Pembrolizumab and Nivolumab, the CTLA-4 mAb Ipilimumab). The insert table shows the major trials targeting this pathway.

Several studies have shown that immunotherapy, reactivating the immune system against tumor cells, could provide a therapeutic advantage in many cancer types [72, 73]. Years ago, some studies proved that potentiating the immune system with cancer vaccines could provide a significant benefit in the adjuvant setting in gastric cancer [74]. Further studies showed the therapeutic potential of adoptive cell therapy with either tumor infiltrating lymphocytes or dendritic cells [75–77]. However, at present, the most promising strategy is immune checkpoint inhibition (Figure 6). Immune checkpoints (involving PD-1 and CTLA-4) are inhibitory pathways crucial for maintaining self-tolerance. PD-1 is a checkpoint protein on T cells that prevents them from attacking cells expressing the PDL1/2 ligands. Monoclonal antibodies that target either PD-1 or PD-L1 can abrogate this checkpoint inhibition, thus boosting the immune response against cancer cells (reviewed in [78]). CTLA-4 (Cytotoxic T-Lymphocyte-Associated protein 4, also called CD152) is constitutively expressed on Treg cells and upregulated in conventional T cells after activation. When bound to CD80 or CD86 it contributes to T lymphocyte inhibitory function [79, 80] Inactivation of these inhibitory checkpoints helps cancer cells to maintain an immunosuppressive microenvironment. Studies performed in other tumor types have shown a remarkable efficacy of drugs targeting these pathways such as Pembrolizumab, Nivolumab (PD-1 mAbs), Avelumab (PDL-1 mAb) and Ipilimumab (CTLA-4 mAb) (reviewed in [81]). Very recently, these drugs have been explored in the contest of gastric cancer as well. The phase I KEYNOTE-012 trial investigated Pembrolizumab in heavily pretreated patients with either recurrent or metastatic tumors of the stomach or the gastro-esophageal junction [82]. Only patients with PD-L1 positive tumors were enrolled; PD-L1 positivity was assessed with immunohistochemistry assays and defined as membrane staining in ≥1% of cells, or for the presence of a distinctive PD-L1 positive pattern at the interface between neoplastic cells and adjacent stroma. Pembrolizumab elicited sustained antitumor responses in 22% of patients (8 /39 patients) by central review, with no significative difference in antitumor activity or safety between Asian or non-Asian patients. The EBV status of the treated patients was not evaluated. Although the data were preliminary, a trend toward an association between higher PD-L1 levels and ORR, PFS and OS was observed. Currently, new trials are investigating the efficacy and safety of Pembrolizumab in patients with advanced gastric cancer. In the MK-3475-059/KEYNOTE-059 Phase II trial (NCT02335411) Pembrolizumab will be given as monotherapy to participants who have had previous treatment or who are treatment-naïve; Pembrolizumab will also be evaluated as combination therapy with chemotherapy in treatment-naïve participants. The randomized, phase III MK-3475-059/KEYNOTE-061 (NCT02370498) will evaluate Pembrolizumab versus paclitaxel in advanced gastric cancer patients with tumor progression after first-line treatment with platinum and fluoropyrimidine doublet therapy. The study hypotheses will primarily evaluate if Pembrolizumab prolongs progression free survival and overall survival and the possible correlation of response with PD-L1 expression. The randomized, phase III MK-3475-059/KEYNOTE-062 trial (NCT02494583) will compare the activity of Pembrolizumab as monotherapy, or Pembrolizumab plus dual chemotherapy, or placebo plus dual chemotherapy, in gastric cancer patients with PD-L1 positive tumors.

Another Phase III trial (NCT02267343), not limiting patient enrollment by PD-L1 biomarker status, was initiated in October 2014 to compare Nivolumab versus placebo in previously treated Japanese patients with unresectable advanced or recurrent cancer. Finally, the randomized, phase III, CheckMate649 trial (NCT02872116) will compare the efficacy of the combination Nivolumab plus Ipilimumab against the chemotherapy standard in gastric cancer patients. Overall survival will be analyzed in all randomized subjects and in subjects with PD-L1 expressing tumors. Initials results for the Phase I/II CheckMate-032 study of Nivolumab monotherapy in advanced and metastatic gastroesophageal cancer patients demonstrated encouraging antitumor activity in heavily pretreated patients (overall response rate 12%: 1 complete response and 6 partial responses; 21% stable disease), regardless from PD-L1 expression [83]. In a Phase Ib study (NCT01772004), Avelumab showed promising clinical activity in unselected patients treated as first-line maintenance or second-line therapy [84], thus supporting the opening of two randomized Phase III trials. Negative results were instead obtained in a Phase II trial (NCT01585987) with Ipilimumab that was stopped post-interim analysis as no difference in immune-related progression free survival was observed versus best supportive care [85]. Finally, with the idea to combine immunotherapy and targeted therapy, two multicenter phase IB/II studies are determining antitumor activity and safety of Pembrolizumab in combination with anti-HER2 agents in patients with HER2 positive gastric cancer (NCT02901301 and NCT02689284).

The available data are certainly at an early stage, but the high response rates and preliminary OS data justify the excitement associated with PD-1 inhibitors and add gastric cancer to the growing list of tumors in which immuno-oncology may play a significant role. The molecular profiling from the TCGA identified elevated PD-L1 expression in the EBV gastric cancer subtype. Although there is no agreement that an increased expression of PD-L1/PD1 is strictly required to obtain a response with the immune checkpoint drugs, it will be interesting to investigate the efficacy of immunotherapy in this subtype of patients. Even more interestingly, will be the evaluation of immunotherapy in patients displaying microsatellite instability. Indeed, a recent work performed on colon cancer showed that the mismatch-repair deficient status predicts clinical benefit of immune checkpoint blockade [88], likely increasing the spectrum of neo-antigens able to elicit an immune response. As around 20% of gastric cancer patients display MSI, they represent a wide subpopulation which could benefit from this therapy.

## CONCLUSIONS

Over the past years, many molecular drugs entered clinical trials for gastric cancer but, with the exception of Trastuzumab and Ramucirumab, all failed. Why did it happen? First of all, despite the high prevalence of gastric cancer, this tumor has been poorly studied from a molecular point of view and only recently few large and comprehensive genomic surveys have been reported. These studies highlighted several molecular alterations in possible driver genes that can thus be considered as promising therapeutic targets. Precision medicine has clearly shown that patient selection for the molecular alteration of the target is mandatory to perform clinical trials likely to define the efficacy of the inhibition of a given target. In this perspective, the study performed by TCGA [6] offers a very powerful instrument to orientate the analysis. For example, tumors of the EBV and MSI subtype are likely to present PI3K mutations while CIN tumors are those displaying the highest frequency of receptor tyrosine kinase amplification.

Another important issue is to define the optimal therapy for the identified target. It is now clear that the tumor context can modify the response to drugs. For example, HER2+ breast cancers respond to Trastuzumab monotherapy while HER2+ colon cancers do not [39, 89]. Moreover, it is not obvious that a mutated gene that behaves as a driver in a context does act similarly in a different tumor, as shown by the V600E BRAF mutants that respond to BRAF inhibitors in 80% of melanomas but only in 5% of colon cancers [90–92]. These observations strongly reinforce the need of an appropriate target validation in the context to be treated. To this purpose, the use of Patients-derived-xenografts (PDX) has been very helpful in different tumor types [93]. Tumor surgical specimens directly transferred in mice, in fact, conserve the inter-individual diversity and the genetic heterogeneity typical of the tumors of origin. Thus, different therapeutic approaches can be tested at the same time in preclinical proof-of concepts trials on cohorts of mice. The system, thus, combines the flexibility of preclinical analysis with the informative value of population-based studies. Different groups are currently generating platforms of gastric PDXs [94, 95] and it is very likely that the data derived by the preclinical work in this system will influence future trials.

Another issue of targeted therapies in gastric cancer is represented by the high degree of intra-tumor heterogeneity frequently displayed by these tumors. This was clearly shown, for example, in HER2 [96] and MET expression [52]. Genetic and molecular heterogeneity can be observed not only inside the tumor, but also at metastatic sites [52], suggesting that an accurate molecular diagnosis should be more and more implemented with liquid biopsy strategies. Ideally, the problem of intra-tumor heterogeneity could be overcome targeting “trunk” mutations, that is somatic alterations driving tumor growth, already present in early clonal progenitors and ubiquitously present at all sites of disease [97]. However, at present, no trunk mutations have been identified in gastric cancer.

Finally, a new prospective is given by the use of immunotherapy. The interesting results recently obtained with the treatment of gastric cancer patients with anti PDL-1 antibodies suggest the therapeutic potential of this approach [82]. Nevertheless, the evaluation of the activity of these drugs in specific molecular subtypes is worthy. In particular, it will be very interesting to investigate if immunotherapies are effective in tumors of the EBV subtype, showing traits of immune system activation, or in those of the MSI subtype, characterized by a high mutational load and a wide spectrum of neo-antigens.

To conclude, we have probably reached a point where the development of targeted therapies in gastric cancer is at a turn. The possibility to combine molecular data, to perform pre-clinical trials on patient-derived material and to design “proof of concept” clinical trials can drive us to a real improvement of the treatment of gastric cancer patients.
